# Impact of Insulin Resistance on Silent and Ongoing Myocardial Damage in Normal Subjects: The Takahata Study

**DOI:** 10.1155/2012/815098

**Published:** 2012-10-10

**Authors:** Taro Narumi, Tetsuro Shishido, Nobuyuki Kiribayashi, Shinpei Kadowaki, Satoshi Nishiyama, Hiroki Takahashi, Takanori Arimoto, Takehiko Miyashita, Takuya Miyamoto, Tetsu Watanabe, Yoko Shibata, Tsuneo Konta, Yoshiyuki Ueno, Takeo Kato, Takamasa Kayama, Isao Kubota

**Affiliations:** ^1^Department of Cardiology, Pulmonology, and Nephrology, Yamagata University School of Medicine, 2-2-2 Iida-Nishi, Yamagata 990-9585, Japan; ^2^Department of Gastroenterology, Yamagata University School of Medicine, Yamagata 990-9585, Japan; ^3^Department of Neurology, Hematology, Metabolism, Endocrinology, and Diabetology, Yamagata University School of Medicine, Yamagata 990-9585, Japan; ^4^Global Center of Excellence Program Study Group, Yamagata University School of Medicine, Yamagata 990-9585, Japan

## Abstract

*Background*. Insulin resistance (IR) is part of the metabolic syndrome (Mets) that develops after lifestyle changes and obesity. Although the association between Mets and myocardial injury is well known, the effect of IR on myocardial damage remains unclear. *Methods and Results*. We studied 2200 normal subjects who participated in a community-based health check in the town of Takahata in northern Japan. The presence of IR was assessed by homeostasis model assessment ratio, and the serum level of heart-type fatty acid binding protein (H-FABP) was measured as a maker of silent and ongoing myocardial damage. H-FABP levels were significantly higher in subjects with IR and Mets than in those without metabolic disorder regardless of gender. Multivariate logistic analysis showed that the presence of IR was independently associated with latent myocardial damage (odds ratio: 1.574, 95% confidence interval 1.1–2.3) similar to the presence of Mets. *Conclusions*. In a screening of healthy subjects, IR and Mets were similarly related to higher H-FABP levels, suggesting that there may be an asymptomatic population in the early stages of metabolic disorder that is exposed to myocardial damage and might be susceptible to silent heart failure.

## 1. Introduction

The presence of metabolic disorders such as glucose intolerance and dyslipidemia is associated with the incidence of cardiovascular disease (CVD) and is a cause of mortality [1, 2]. It has been reported that the components of metabolic syndrome (Mets), which include abdominal obesity, hypertension, impaired insulin tolerance with high fasting glucose levels, and elevated levels of triglycerides [3–5], are risk factors for CVD [6, 7]. Epidemiological and experimental studies have provided evidence of the relationship between cardiac dysfunction and diabetes mellitus (DM) [8–11]. Furthermore, hyperglycemia, hypertension, and dyslipidemia are associated with ongoing myocardial damage. These findings imply that there is a significant association between the severity of Mets and organ damage [12, 13]. However, a correlation between insulin resistance (IR) and cardiac dysfunction in the general population has not been established to date.

Clinical studies have shown that heart-type fatty acid-binding protein (H-FABP), which is rapidly released into the circulation from the damaged myocardium, may be a marker for myocardial damage not only in patients with ischemic heart disease but also in those with chronic heart failure [14–17]. Because the levels of H-FABP are correlated with the incidence of cardiac events in heart failure patients, the assessment of H-FABP levels may be of value to estimate the potential existence of cardiac damage in the general population [18, 19]. The effect of IR on myocardial damage in normal subjects, however, remains to be clarified. The purpose of this study was to investigate the association between IR and myocardial damage in healthy subjects.

## 2. Methods

### 2.1. Study Design

This study was part of the Molecular Epidemiological Study utilizing the Regional Characteristics of 21st Century Centers of Excellence (COE) Program and Global COE Program in Japan, as described in detail previously [20]. The study was approved by the institutional ethics committee and all participants provided written informed consent. The subjects included in the study were members of the general population with an age of 40 years and older from the town of Takahata in northern Japan. From June 2004 to November 2005, 1,380 men and 1,735 women were enrolled in the study. A total of 915 patients with incomplete data were excluded, and 2200 patients participated in the final study.

### 2.2. Clinical Assessments

The Takahata town study was based on a survey consisting of a self-administered questionnaire about lifestyle, blood pressure measurements, anthropometric measurements, and the collection of blood and urine specimens from the participants at annual health exams. Information concerning medical history, current medications, smoking habits, and alcohol intake was obtained from the self-reported questionnaire. Blood pressure was measured using a mercury manometer, with subjects resting in a seated position for at least 5 minutes before measurement. 

### 2.3. Definition of Mets and IR

Mets was evaluated using the National Cholesterol Education program Adult Treatment Panel III (NCEP-ATP III) criteria [3]. The NCEP-ATP III criteria for abdominal obesity were modified by using body mass index (BMI) ≥ 25 kg/m^2^ instead of the waist circumference because obesity is defined as BMI ≥ 25 kg/m^2^ in Japan [21, 22]. Mets was defined on the basis of meeting at least 3 of the following 5 NCEP-ATP III criteria: BMI ≥ 25 kg/m^2^, elevated triglyceride (TG) ≥ 150 mg/dL, reduced high-density lipoprotein cholesterol (HDLc) < 40 mg/dL in men and < 50 mg/dL in women, elevated fasting blood sugar (FBS) ≥ 110 mg/dL or previously diagnosed diabetes mellitus, elevated blood pressure [systolic blood pressure (sBP) ≥ 130 mmHg, and/or diastolic blood pressure (dBP) ≥ 85 mmHg] or use of antihypertensive medication. Insulin tolerance was evaluated with the homeostasis model assessment ratio [HOMA-R, HOMA-R = fasting insulin levels (IRI) × FBS × 1/405], and IR was defined as HOMA-R > 2.5 [23]. 

### 2.4. Definition of Latent and Ongoing Myocardial Damage

The presence of latent and ongoing myocardial damage was defined as serum levels of heart type fatty acid binding protein (H-FABP) above 4.3 ng/mL as reported previously [16, 18]. 

### 2.5. Statistical Analysis

Data are presented as mean ± standard deviation (SD). Data that were not distributed normally were presented as medians and interquartile intervals. The unpaired Student's *t*-test and the chi-square test were used for comparisons between 2 groups of continuous and categorical variables, respectively. The Mann-Whitney *U*-test was used when data were not distributed normally. Comparisons of data among 3 groups categorized based on the presence of IR and Mets were performed by the Kruskal-Wallis test. Univariate and multivariate logistic analyses were performed to evaluate the association between IR and ongoing myocardial damage.

## 3. Results

### 3.1. Patient Characteristics

Table 1 lists the characteristics of the 2200 subjects. The proportion of men was 44.2%, and the mean age of the study subjects was 63 ± 10 years. Mean serum levels of brain natriuretic peptide (BNP) and H-FABP were 20.0 ng/L (interquartile range: 10.9–35.8) and 3.5 ng/mL (interquartile range: 2.6–4.7), respectively. Mean BMI was 23.0 ± 3.3 kg/m^2^; sBP and dBP were 134 ± 16 mmHg and 79 ± 10 mmHg, respectively. Serum FBS levels were 93 ± 12 mg/dL. Serum levels of total cholesterol (TC), TG, HDLc, and low-density lipoprotein cholesterol (LDLc) were 200 mg/dL (interquartile range: 179–221), 91 mg/dL (interquartile range: 68–125), 59 ± 15 mg/dL, and 124 ± 29 mg/dL, respectively. Estimated glomerular filtration rate was 96.0 mL/min/1.73 m^2^ (interquartile range: 82–110).

### 3.2. Classification of Subjects by the Presence of IR

**Figure 1:**
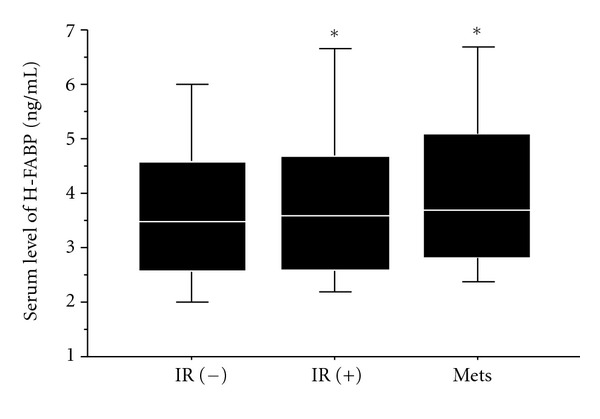
Serum levels of H-FABP in the study population. Subjects with and without insulin resistance (IR) and metabolic syndrome (Mets) were included. In comparison to IR (−) subjects, subjects with IR and Mets showed an increase in H-FABP levels. Data are expressed as mean ± SD. *P < 0.05 versus subjects without IR.

Subjects were divided into 3 groups according to the presence of IR and Mets, as shown in Table 2. IR was associated with female gender; high BMI; high sBP and dBP; high LDLc, TG, and TC levels; and low BNP and HDLc levels compared to subjects without IR. Mets was associated with male gender; high BMI; high sBP and dBP; high LDLc, TG, and TC levels; and low BNP and HDLc levels compared to subjects without Mets. Serum H-FABP levels were significantly higher in subjects with IR and Mets than in those without IR. However, there was no significant difference between subjects with IR and those with Mets, as shown in Figure 1.

### 3.3. Classification by Gender

It is well known that serum H-FABP level and other blood parameters differ according to gender [18]. We therefore divided subjects into 2 groups according to gender, as shown in Table 3. Clinical characteristics were different between the 2 groups. However, serum H-FABP levels were significantly higher in subjects with IR and Mets than in those without IR in both groups regardless of gender, as shown in Tables 4 and 5.

### 3.4. Association between IR and Ongoing Myocardial Damage

**Figure 2:**
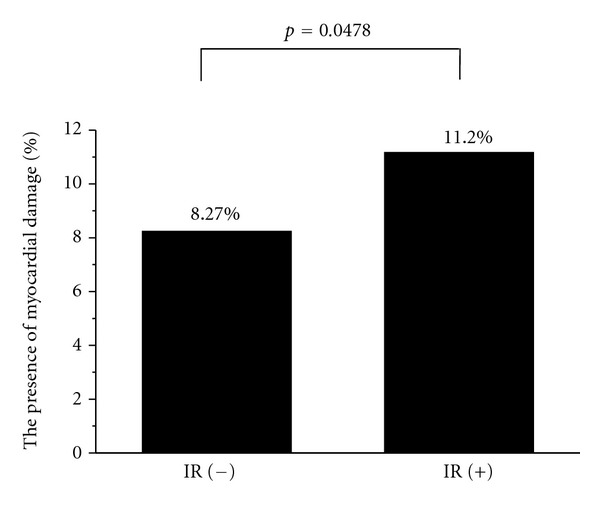
Comparison of latent and ongoing myocardial damage between subjects with and without IR. Latent and ongoing myocardial damage was defined as serum levels of heart type fatty acid binding protein (H-FABP) above 4.3 ng/mL. In comparison to IR (−) subjects, those with IR showed a higher incidence of myocardial damage.

To investigate the contribution of IR to the increase of H-FABP levels in the asymptomatic general population, 1936 subjects without Mets were examined by univariate and multivariate logistic analyses. In the univariate logistic analysis, the presence of IR, age, gender, sBP, and serum levels of BNP and LDLc were associated with latent and ongoing myocardial damage, as shown in Table 6. In the multivariate logistic analysis, the presence of IR was independently associated with the increase of H-FABP levels (OR: 1.6, 95% confidence interval 1.1–2.3) after adjusting for age, gender, serum BNP level, sBP, and serum LDLc level, as shown in Table 3. Furthermore, the percentage of subjects with latent and ongoing myocardial damage was significantly higher in those with IR than in those without IR (8.27% versus 11.20%, *P* = 0.0478), as shown in Figure 2.

## 4. Discussion

The findings of the present study are as follows: (1) H-FABP levels increased in association with IR and Mets in normal subjects; (2) multivariate logistic analysis revealed that the presence of IR was an independent risk factor for myocardial injury in the general population; (3) the prevalence of high H-FABP was more prevalent in subjects with IR than in those without IR.

Although patients with IR are often asymptomatic, this condition can lead to a multitude of diseases [24]. The early detection of metabolic disorder is important for the prevention of new-onset CVD. Physicians can only treat patients in the symptomatic state because asymptomatic subjects, such as those with IR, do not generally present to clinics or hospitals. Our study showed that latent and ongoing myocardial damage may occur in the early stages of metabolic disease, such as those characterized by the presence of IR. This suggests that early detection of latent and ongoing myocardial damage through population screening is essential to prevent future cardiovascular disorders.

Serum BNP is an established and commonly used marker for the detection of myocardial damage during screening evaluations [25]. However, in the present study, we measured serum H-FABP to detect latent and ongoing myocardial damage for the following reasons. First, several cohort studies have shown that high BMI is inversely correlated to serum BNP level [26–28]. The hypothesized mechanism underlying this inverse relationship has been described previously. Increased expression of natriuretic peptide clearance receptor by adipose tissue, which is shown in obese subjects, results in increased clearance of serum BNP [29]. Similarly, we also observed an inverse relationship between serum BNP level and IR in the present study. Thus, serum BNP appears to be inadequate to detect latent and ongoing myocardial damage in the early stages of metabolic disorder. Second, previous reports showed that serum H-FABP is a useful and sensitive marker for screening patients with latent and ongoing myocardial damage [14, 17, 19]. It is well known that serum H-FABP is rapidly released into the circulation from damaged myocardium; hence, it is used as a marker of acute coronary syndrome. On the other hand, there is evidence that disturbances in cardiac microvascular circulation caused by metabolic disorders and left ventricular hypertrophy induce myocardial hypoxia and cardiomyocyte injury and impair cardiac function [30–32]. Moreover, not only hypoxia, but also mechanical stretch, oxidative stress, inflammation, and apoptosis increase cardiomyocyte permeability, resulting in elevated levels of myocardial cytosolic markers in patients with heart failure [33–36]. Previously, we demonstrated that increased serum H-FABP level is associated with the exacerbation of chronic heart failure and thus provides prognostic information [15, 16]. These studies showed that patients with high serum levels of H-FABP had a significantly higher rate of cardiac events than those with normal H-FABP levels (34% versus 4%, *P* < 0.001). Furthermore, these studies suggested that serum H-FABP level was an independent predictor of future cardiac events (*χ* ^2^ = 7.397, *P* < 0.01). Similarly, minimally increased levels of troponin T are associated with mortality and morbidity in patients with chronic heart failure [37, 38]. 

In this study, we demonstrated that H-FABP but not BNP was higher in subjects with IR and Mets than in those without these conditions, suggesting that measurement of H-FABP might be suitable to predict the occurrence of myocardial damage in subjects with metabolic disorder.

There were several limitations associated with our study. First, we modified the NCEP-ATP III criteria for abdominal obesity by using body mass index (BMI) ≥ 25 kg/m^2^ instead of the waist circumference to evaluate metabolic syndrome. We used BMI instead of the waist circumference because the definition of waist circumference is unclear in Japan, especially in women [39]. On the other hand, BMI as a criterion for obesity has been clearly defined as BMI ≥ 25 kg/m^2^ in the Japanese general population [21]. Second, we did not detect latent and ongoing myocardial damage by visual methods such as echocardiography or myocardial scintigraphy. The association between alterations in diastolic function and metabolic disorder has been reported [40], and the correlation between the results of myocardial scintigraphy and myocardial damage in subjects with diastolic dysfunction was also been shown [41]. In addition, prior reports have shown that H-FABP levels are correlated with the severity of myocardial injury evaluated by myocardial scintigraphy [42]. Taken together, these findings suggest that the measurement of serum levels of H-FABP might be appropriate for a population-based study. Third, our data are cross-sectional and cannot demonstrate longitudinal progression of cardiovascular disorder and cannot establish causal relationship between IR and obvious heart disease. Future studies should be focused on assessing longitudinal progression.

## 5. Conclusion

The presence of IR is related to latent and ongoing myocardial damage in normal subjects, which suggests that myocardial damage occurs in the early state of metabolic disorder. Future studies should be aimed at developing effective strategies for the treatment of IR to prevent myocardial damage and improve clinical outcomes.

## Figures and Tables

**Table 1:** Comparisons of clinical characteristics of 2200 subjects.

Age (years)*	69.1 ± 12.6
Gender (male/female)	973/1227
BMI (kg/m^2^)*	23.0 ± 3.3
sBP (mm Hg)*	134 ± 16
dBP (mm Hg)*	79 ± 10
BNP (pg/mL)**	20.0 (10.9–35.8)
TC (mg/dL)**	200 (179–221)
TG (mg/dL)**	91 (68–125)
HDLc (mg/dL)*	59 ± 15
LDLc (mg/dL)*	124 ± 29
eGFR (mL/min/1.73 m^2^)**	96 (82–110)
H-FABP (ng/mL)**	3.5 (2.6–4.7)

BMI: body mass index; BNP: brain natriuretic peptide; TC: total cholesterol; TG: triglyceride; HDLc: high density lipoprotein cholesterol; LDLc: low density lipoprotein cholesterol; H-FABP: heart type fatty acid binding protein; sBP: systolic blood pressure; dBP: diastolic blood pressure; Data are presented as *mean ± S.D; **median (interquartile range).

**Table 2:** Comparisons of clinical characteristics of patients with and without IR.

	IR (−)	IR (+)	Mets
Age	63 ± 10	63 ± 10	63 ± 10
Male	659 (46.2%)	169 (33.2%)*	145 (54.9%)^∗#^
BMI	21.9 ± 2.8	24.4 ± 3.2*	26.1 ± 2.5^∗#^
sBP	132 ± 16	136 ± 15*	134 ± 14*
dBP	78 ± 10	80 ± 10*	84 ± 8^∗#^
BNP (pg/mL)	21.5 (12.1–37.4)	17.1 (9.5–30.5)*	17.5 (9.4–33.6)*
TC (g/dL)	197 (177–217)	204 (187–226)*	205 (182–229)*
TG (g/dL)	81 (63–106)	101 (77–128)*	171 (150–224)^∗#^
HDL (g/dL)	61 ± 14	58 ± 13*	46 ± 12^∗#^
LDL (g/dL)	122 ± 29	131 ± 28*	124 ± 30^∗#^
eGFR (mL/min/1.73 m^2^)	97 (82–112)	98 (83–111)	86 (74–102)^∗#^
H-FABP (ng/mL)	3.5 (2.6–4.6)	3.6 (2.6–4.7)*	3.7 (2.8–5.1)*

BMI: body mass index; BNP: brain natriuretic peptide; TC: total cholesterol; TG: triglyceride; HDLc: high density lipoprotein cholesterol; LDLc: low density lipoprotein cholesterol; H-FABP: heart type fatty acid binding protein; sBP: systolic blood pressure; dBP: diastolic blood pressure; Data are presented as mean ± S.D or median (interquartile range). *P < 0.05 versus IR (−); ^#^ *P* < 0.05 versus IR (+).

**Table 3:** Comparisons of clinical characteristics in male and female subjects.

	Male	Female
Age (years)*	64.0 ± 10.2	62.7 ± 10.0^†^
BMI (kg/m^2^)*	22.9 ± 3.0	23.1 ± 3.5
sBP (mm Hg)*	136 ± 16	133 ± 16^†^
dBP (mm Hg)*	82 ± 10	77 ± 9^†^
BNP (pg/mL)**	17.6 (9.3–36.1)	21.4 (12.4–35.3)
TC (mg/dL)**	191 (173–211)	206 (187–226)^†^
TG (mg/dL)**	95 (70–135)	89 (66–117)^†^
HDLc (mg/dL)*	56 ± 14	61 ± 14^†^
LDLc (mg/dL)*	119 ± 28	129 ± 28^†^
eGFR (mL/min/1.73 m^2^)**	82 (72–90)	106 (97–124)^†^
H-FABP (ng/mL)**	3.7 (2.8–4.8)	3.4 (2.6–4.6)^†^

BMI: body mass index; BNP: brain natriuretic peptide; TC: total cholesterol; TG: triglyceride; HDLc: high density lipoprotein cholesterol; LDLc: low density lipoprotein cholesterol; H-FABP: heart type fatty acid binding protein; sBP: systolic blood pressure; dBP: diastolic blood pressure; Data are presented as *mean ± S.D; **median (interquartile range); ^†^ *P* < 0.05 versus male.

**Table 4:** Comparisons of clinical characteristics of patients with and without IR and Mets in male.

	IR (−)	IR (+)	Mets (+)
Age (years)	64 ± 10	64 ± 10	63 ± 8
BMI (kg/m^2^)	22.0 ± 3.0	24.2 ± 2.8*	25.9 ± 2.3^∗#^
sBP (mm Hg)	135 ± 16	138 ± 16*	142 ± 13*
dBP (mm Hg)	81 ± 10	83 ± 10	85 ± 9*
BNP (pg/mL)	19.8 (10.2–37.6)	13.8 (8.0–31.8)*	14.2 (8.0–28.7)*
TC (mg/dL)	189 (170–207)	197 (179–215)*	196 (173–224)^∗#^
TG (mg/dL)	84 (64–111)	104 (79–135)*	184 (151–256)*
HDLc (mg/dL)	59 ± 14	54 ± 12*	43 ± 12^∗#^
LDLc (mg/dL)	117 ± 28	128 ± 27*	123 ± 29^∗#^
eGFR (mL/min/1.73 m^2^)	83 (72–92)	76 (66–86)*	77 (67–89)*
H-FABP (ng/mL)	3.2 (2.4–4.5)	3.6 (2.7–4.8)*	3.8 (2.8–4.9)*

BMI: body mass index; BNP: brain natriuretic peptide; TC: total cholesterol; TG: triglyceride; HDLc: high density lipoprotein cholesterol; LDLc: low density lipoprotein cholesterol; H-FABP: heart type fatty acid binding protein; sBP: systolic blood pressure; dBP: diastolic blood pressure; Data are presented as mean ± S.D or median (interquartile range). *P < 0.05 versus IR (−); ^#^ *P* < 0.05 versus IR (+).

**Table 5:** Comparisons of clinical characteristics of patients with and without IR and Mets in female.

	IR (−)	IR (+)	Mets (+)
Age (years)	62 ± 10	62 ± 10	64 ± 8
BMI (kg/m^2^)	22.0 ± 3.0	24.5 ± 3.4*	26.4 ± 2.7^∗#^
sBP (mm Hg)	130 ± 16	135 ± 15*	144 ± 12^∗#^
dBP (mm Hg)	75 ± 9	78 ± 9*	83 ± 8^∗#^
BNP (pg/mL)	23.3 (14.0–37.0)	18.5 (10.8–30.5)*	19.8 (10.7–38.0)*
TC (mg/dL)	204 (185–223)	209 (190–232)*	212 (192–237)*
TG (mg/dL)	79 (62–102)	100 (76–123)*	166 (149–186)^∗#^
HDLc (mg/dL)	64 ± 14	61 ± 13*	48 ± 11^∗#^
LDLc (mg/dL)	127 ± 28	133 ± 29*	132 ± 29*
eGFR (mL/min/1.73 m^2^)	107 (98–126)	104 (96–123)	101 (86–121)*
H-FABP (ng/mL)	3.3 (2.5–4.4)	3.6 (2.6–4.8)*	3.5 (2.7–5.2)*

BMI: body mass index; BNP: brain natriuretic peptide; TC: total cholesterol; TG: triglyceride; HDLc: high density lipoprotein cholesterol; LDLc: low density lipoprotein cholesterol; H-FABP: heart type fatty acid binding protein; sBP: systolic blood pressure; dBP: diastolic blood pressure; Data are presented as mean ± S.D or median (interquartile range). *P < 0.05 versus IR (−); ^#^ *P* < 0.05 versus IR (+).

**Table 6:** Univariate and multivariate logistic analysis for high serum level of H-FABP.

Variables	OR	95% CI	*P* value
Univariable analysis			
Age (per 5 years increase)	1.716	1.552–1.908	<0.0001
Male	1.396	1.022–1.905	0.0357
BMI	1.224	0.881–1.701	0.2288
BNP	1.004	1.002–1.006	0.0008
sBP	1.493	1.076–2.071	0.0164
dBP	1.110	0.784–1.570	0.5565
LDLc	0.682	0.468–0.995	0.0468
HDL (<40 mg/dL in male, <50 mg/dL in female)	1.532	0.774–3.035	0.2209
TG (above 150 mg/dL)	0.730	0.377–1.415	0.3514
TC (above 220 mg/dL)	0.960	0.670–1.377	0.8265
IR (presence of IR)	1.399	1.002–1.953	0.0487
Multivariable analysis			
Age (per 5 years increase)	1.707	1.531–1.908	<0.0001
Male	1.258	0.904–1.750	0.1726
BNP	1.022	0.719–1.454	0.9023
sBP	1.041	0.736–1.472	0.8219
IR (presence of IR)	1.574	1.100–2.251	0.0131

OR: odds ratio; CI: confidence interval; BMI: body mass index; BNP: brain natriuretic peptide; TC: total cholesterol; TG: triglyceride; HDLc: high density lipoprotein cholesterol; LDLc: low density lipoprotein cholesterol; H-FABP: heart type fatty acid binding protein; sBP: systolic blood pressure; dBP: diastolic blood pressure.

## References

[1] Lucove J, Vupputuri S, Heiss G, North K, Russell M (2008). Metabolic syndrome and the development of CKD in American Indians: the Strong Heart Study. *American Journal of Kidney Diseases*.

[2] Hassan SA, Deswal A, Bozkurt B, Aguilar D, Mann DL, Pritchett AM (2008). The metabolic syndrome and mortality in an ethnically diverse heart failure population. *Journal of Cardiac Failure*.

[3] Cleeman JI (2001). Executive summary of the third report of the National Cholesterol Education Program (NCEP) expert panel on detection, evaluation, and treatment of high blood cholesterol in adults (adult treatment panel III). *Journal of the American Medical Association*.

[4] Alexander CM, Landsman PB, Teutsch SM, Haffner SM (2003). NCEP-defined metabolic syndrome, diabetes, and prevalence of coronary heart disease among NHANES III participants age 50 years and older. *Diabetes*.

[5] Matsuzawa Y (2005). Metabolic syndrome–definition and diagnostic criteria in Japan. *Journal of Atherosclerosis and Thrombosis*.

[6] Noda H, Iso H, Saito I, Konishi M, Inoue M, Tsugane S (2009). The impact of the metabolic syndrome and its components on the incidence of ischemic heart disease and stroke: the Japan public health center-based study. *Hypertension Research*.

[7] Bjorntorp P (1990). ’Portal’ adipose tissue as a generator of risk factors for cardiovascular disease and diabetes. *Arteriosclerosis*.

[8] Shishido T, Woo CH, Ding B (2008). Effects of MEK5/ERK5 association on small ubiquitin-related modification of ERK5: implications for diabetic ventricular dysfunction after myocardial infarction. *Circulation Research*.

[9] Horwich TB, Fonarow GC (2010). Glucose, obesity, metabolic syndrome, and diabetes. Relevance to incidence of heart failure. *Journal of the American College of Cardiology*.

[10] Le NT, Takei Y, Shishido T, Woo CH, Chang E, Heo KS (2012). p90RSK targets the ERK5-CHIP ubiquitin E3 ligase activity in diabetic hearts and promotes cardiac apoptosis and dysfunction. *Circulation Research*.

[11] Woo CH, Le NT, Shishido T (2010). Novel role of C terminus of Hsc70-interacting protein (CHIP) ubiquitin ligase on inhibiting cardiac apoptosis and dysfunction via regulating ERK5-mediated degradation of inducible cAMP early repressor. *The FASEB Journal*.

[12] Lakka HM, Laaksonen DE, Lakka TA (2002). The metabolic syndrome and total and cardiovascular disease mortality in middle-aged men. *Journal of the American Medical Association*.

[13] Rubin J, Matsushita K, Ballantyne CM, Hoogeveen R, Coresh J, Selvin E (2012). Chronic hyperglycemia and subclinical myocardial injury. *Journal of the American College of Cardiology*.

[14] Setsuta K, Seino Y, Ogawa T, Arao M, Miyatake Y, Takano T (2002). Use of cytosolic and myofibril markers in the detection of ongoing myocardial damage in patients with chronic heart failure. *American Journal of Medicine*.

[15] Arimoto T, Takeishi Y, Shiga R (2005). Prognostic value of elevated circulating heart-type fatty acid binding protein in patients with congestive heart failure. *Journal of Cardiac Failure*.

[16] Arimoto T, Takeishi Y, Niizeki T (2005). Ongoing myocardial damage relates to cardiac sympathetic nervous disintegrity in patients with heart failure. *Annals of Nuclear Medicine*.

[17] Morioka N, Shigematsu Y, Hamada M, Higaki J (2005). Circulating levels of heart-type fatty acid-binding protein and its relation to thallium-201 perfusion defects in patients with hypertrophic cardiomyopathy. *American Journal of Cardiology*.

[18] Niizeki T, Takeishi Y, Takabatake N (2007). Circulating levels of heart-type fatty acid-binding protein in a general Japanese population—effects of age, gender and physiologic characteristics. *Circulation Journal*.

[19] Setsuta K, Seino Y, Ogawa T, Ohtsuka T, Seimiya K, Takano T (2004). Ongoing myocardial damage in chronic heart failure is related to activated tumor necrosis factor and Fas/Fas ligand system. *Circulation Journal*.

[20] Konta T, Hao Z, Abiko H (2006). Prevalence and risk factor analysis of microalbuminuria in Japanese general population: the Takahata study. *Kidney International*.

[21] (2002). New criteria for 'obesity disease' in Japan. *Circulation Journal*.

[22] Shishido T, Konta T, Nishiyama S (2011). Suppressive effects of valsartan on microalbuminuria and CRP in patients with metabolic syndrome (Val-Mets). *Clinical and Experimental Hypertension*.

[23] Matthews DR, Hosker JP, Rudenski AS (1985). Homeostasis model assessment: insulin resistance and *β*-cell function from fasting plasma glucose and insulin concentrations in man. *Diabetologia*.

[24] Bloomgarden ZT (2007). Insulin resistance concepts. *Diabetes Care*.

[25] Kitahara T, Shishido T, Suzuki S (2010). Serum midkine as a predictor of cardiac events in patients with chronic heart failure. *Journal of Cardiac Failure*.

[26] Beleigoli A, Diniz M, Nunes M, Barbosa M, Fernandes S, Abreu M (2011). Reduced brain natriuretic peptide levels in class III obesity: the role of metabolic and cardiovascular factors. *Obesity Facts*.

[27] Clerico A, Giannoni A, Vittorini S, Emdin M (2012). The paradox of low BNP levels in obesity. *Heart Failure Reviews*.

[28] Wang TJ, Larson MG, Levy D (2004). Impact of obesity on plasma natriuretic peptide levels. *Circulation*.

[29] Das SR, Drazner MH, Dries DL (2005). Impact of body mass and body composition on circulating levels of natriuretic peptides: results from the Dallas Heart Study. *Circulation*.

[30] Marcus ML, Koyanagi S, Harrison DG (1983). Abnormalities in the coronary circulation that occur as a consequence of cardiac hypertrophy. *American Journal of Medicine*.

[31] Sano M, Minamino T, Toko H (2007). p53-induced inhibition of Hif-1 causes cardiac dysfunction during pressure overload. *Nature*.

[32] Yoon YS, Uchida S, Masuo O (2005). Progressive attenuation of myocardial vascular endothelial growth factor expression is a seminal event in diabetic cardiomyopathy: restoration of microvascular homeostasis and recovery of cardiac function in diabetic cardiomyopathy after replenishment of local vascular endothelial growth factor. *Circulation*.

[33] Prabhu SD (2004). Cytokine-induced modulation of cardiac function. *Circulation Research*.

[34] Robinson AD, Ramanathan KB, McGee JE, Newman KP, Weber KT (2011). Oxidative stress and cardiomyocyte necrosis with elevated serum troponins: pathophysiologic mechanisms. *American Journal of the Medical Sciences*.

[35] Davies CH, Harding SE, Poole-Wilson PA (1996). Cellular mechanisms of contractile dysfunction in human heart failure. *European Heart Journal*.

[36] Olivetti G, Abbi R, Quaini F (1997). Apoptosis in the failing human heart. *New England Journal of Medicine*.

[37] Sato Y, Yamada T, Taniguchi R (2001). Persistently increased serum concentrations of cardiac troponin T in patients with idiopathic dilated cardiomyopathy are predictive of adverse outcomes. *Circulation*.

[38] Egstrup M, Schou M, Tuxen CD, Kistorp CN, Hildebrandt PR, Gustafsson F (2012). Prediction of outcome by highly sensitive troponin T in outpatients with chronic systolic left ventricular heart failure. *American Journal of Cardiology*.

[39] Hara K, Matsushita Y, Horikoshi M (2006). A proposal for the cutoff point of waist circumference for the diagnosis of metabolic syndrome in the Japanese population. *Diabetes Care*.

[40] Powell BD, Redfield MM, Bybee KA, Freeman WK, Rihal CS (2006). Association of obesity with left ventricular remodeling and diastolic dysfunction in patients without coronary artery disease. *American Journal of Cardiology*.

[41] Katoh S, Shishido T, Kutsuzawa D (2010). Iodine-123-metaiodobenzylguanidine imaging can predict future cardiac events in heart failure patients with preserved ejection fraction. *Annals of Nuclear Medicine*.

[42] Arimoto T, Takeishi Y, Niizeki T (2007). Cardiac sympathetic denervation and ongoing myocardial damage for prognosis in early stages of heart failure. *Journal of Cardiac Failure*.

